# Effect of Curvature Height on the Low-Velocity Impact Behaviour of Unidirectional T300/5208 CFRP Laminated Shell Panels: A Comparative Numerical Parametric Analysis of Intralaminar + Interlaminar and Intralaminar-Only Models

**DOI:** 10.3390/polym18111290

**Published:** 2026-05-24

**Authors:** Onur Gök

**Affiliations:** Department of Machine and Metal Technologies, Seydişehir Vocational School, Necmettin Erbakan University, 42370 Seydişehir, Konya, Türkiye; ogok@erbakan.edu.tr

**Keywords:** low-velocity impact, CFRP shell panel, curvature height, cohesive interface, delamination, CSDMG, comparative model, Hashin damage model, SC8R continuum shell, impact performance index, surface-area expansion ratio

## Abstract

In this study, the 33.5 J low-velocity impact (LVI) behaviour of unidirectional T300/5208 CFRP cylindrical shell panels with a 40-ply [45/0/−45/90]_5s_ layup was investigated using Abaqus/Explicit under the effect of the curvature-height parameter (f = 0–62.5 mm; a_1_–a_6_). To address the limitation of the previous single-block approach in not being able to represent delamination, the study was carried out on two models: an intralaminar-only (SC8R single-block) model and an intralaminar + interlaminar model containing nine cohesive interfaces. Quantitative results: In the intralaminar-only model, the maximum contact force peaks at a_3_ (f = 25 mm), with 13,192 N, representing a 13.7% increase relative to the flat panel; whereas in the intralaminar + interlaminar model, the force is highest at a_2_ (f = 12.5 mm), with 14663 N, and decreases monotonically with curvature (10,765 N at a_6_). Failure mechanism: In the intralaminar-only model, the dominant intralaminar mode is matrix tensile damage (DAMAGEMT); in the intralaminar + interlaminar model, interlaminar separation (CSDMG) governs the total damage, and the initiated delamination area reaches its minimum at a_4_ (f = 37.5 mm), with 7282 mm^2^, and its maximum at a_5_, with 9821 mm^2^. Thus, a curvature-dependent delamination-minimum regime arises that differs from the a_3_ optimum of the intralaminar-only model. An impact performance index (DPI) and its surface-area-corrected derivative, DPI* = DPI/ζ, were applied separately for both models. It was shown that delamination systematically lowers the performance level and shifts the optimum curvature window. All findings are comparative trends within a single numerical framework.

## 1. Introduction

Carbon-fibre-reinforced polymer (CFRP) composites are increasingly used in the aerospace, defence, marine and automotive sectors owing to their high specific stiffness, superior corrosion resistance and design flexibility [1,2]. Compared with glass-fibre (GFRP)- and basalt-fibre (BFRP)-reinforced polymers, carbon-fibre composites offer markedly higher specific strength and specific modulus. The tensile modulus of CFRP is typically about 3–5 times higher than that of GFRP and 2–3 times higher than that of BFRP, and its density (~1.54 g/cm^3^) is lower than those of GFRP (~2.5 g/cm^3^) and BFRP (~2.7 g/cm^3^) [3]. In addition, CFRP exhibits superior fatigue life under cyclic loading and higher durability under moist/thermal ageing conditions. These mechanical, fatigue and durability advantages make CFRP the preferred choice for weight-critical primary structures [4]. Beyond impact loading, the long-term durability of CFRP under hygrothermal and freeze–thaw exposure remains a critical research topic for primary structures [3], and hybrid carbon–glass reinforcement strategies have been investigated to enhance the chemical durability of FRP elements in service environments [4]. Conversely, the low failure strain of carbon fibre and its brittle matrix behaviour make CFRP more susceptible to damage under impact than GFRP/BFRP. This makes a careful investigation of the impact response of CFRP indispensable.

In primary structural applications, such as fuselage panels, wing skins and turbine blades, the most critical weak point of these materials is low-velocity impact (LVI) loading [5]. LVI events produce embedded damage mechanisms that can easily be missed during surface inspection but significantly reduce the residual compressive strength. This has been emphasized in early experimental studies investigating the response of laminated composite plates under impact [6].

Impact damage in composites results from the interaction of three interrelated fundamental mechanisms: (i) intralaminar matrix cracking and fibre breakage, (ii) interlaminar delamination and (iii) back-face fibre rupture. Experimental non-destructive methods such as microscopic cross-section examinations, ultrasonic C-scan and X-ray tomography, together with digital image correlation analyses [7], have shown that delamination mostly develops in a fir-tree pattern depending on the angular mismatch between the fibre orientations of adjacent plies, and that matrix cracks act as delamination initiation points. On the theoretical/numerical side, intralaminar damage is represented by stress-based criteria such as the Hashin criterion [8] and by energy-based evolution models such as the Lapczyk–Hurtado model [9], while interlaminar delamination is represented by cohesive zone models (CZM) and virtual crack closure techniques. The joint modelling of these two damage families is regarded as critical for correctly establishing the impact energy balance [10,11].

The analysis of composites using the finite element method (FEM) has matured over the last two decades. The transfer of nonlinear material behaviour, damage onset and evolution models into the numerical environment has become possible [12]. In intralaminar damage modelling, the Hashin criterion [8] and the Lapczyk–Hurtado energy-based linear evolution model [9] are widely accepted. SC8R continuum-shell elements are preferred in LVI analyses of thin laminates as they balance computational efficiency with accuracy [13]. In terms of fracture-energy parameters, the values measured by Pinho et al. [14] within the framework of NASA/TM-2005-213530 and the standard elastic/strength data of Tsai and Hahn [15] have been adopted as reference data in the literature.

In the numerical prediction of the LVI response, the comparative evaluation of different modelling approaches stands out. Studies that systematically examine Hashin/Puck criteria, instantaneous/linear/exponential evolution methods and cohesive element types on thin CFRP laminates have shown that the damage response is more sensitive to the evolution method than to the initiation criterion [16,17]. Physically sound impact simulations [11] and progressive failure models [17] have been developed in this direction. The modelling of interlaminar delamination with cohesive interface elements has been examined by Aymerich et al. [18], Soto et al. [19], Bouvet et al. [20] and Liu et al. [21], while the effect of dispersed stacking sequences has been examined by Lopes et al. [22]. Drop-weight impact and compression-after-impact (CAI) simulations with cohesive-zone modelling [23,24,25], impact on preloaded panels [26,27] and continuum-damage-mechanics-based plate analyses [28,29,30,31,32] also occupy a wide place in the literature. Plasticity-coupled damage in three-dimensional fibre-reinforced composites [33], the effect of sandwich core thickness [34] and experimental/numerical investigations of the damage process in CFRP beams [35] are also among the related studies. In a broader context, studies such as the quasi-static response of additively manufactured structures [36], the probabilistic modelling of multiple surface-crack propagation [37] and the multi-scale damage modelling of nanocomposites [38] also complement the damage modelling literature. Efficient approaches for quantitative damage measurement have been proposed by Esrail and Kassapoglou [39]. For a comprehensive review of the numerical modelling of interlaminar and intralaminar mechanisms, reference can be made to Taherzadeh-Fard et al. [10].

Although it is known that curved shell geometries significantly change the LVI response, studies addressing this effect through a systematic parametric scan of the curvature height are limited. The validation of different stacking sequences in LVI modelling [40] and stacking-dependent delamination distributions [22] have shown that the stacking sequence significantly changes the response.

This literature review reveals three gaps: (i) parametric studies that systematically scan the curvature height are limited, and dimensionless performance metrics for the curvature–force/energy relationship have not been sufficiently developed; (ii) the majority of existing curved-panel studies address either only intralaminar damage or only delamination, while a direct and quantitative comparison of intralaminar + interlaminar and intralaminar-only models on the same parametric geometry family has not been carried out; and (iii) the effect of the surface-area (mass) increase accompanying high-curvature configurations on the apparent gain in impact response has not been clearly isolated in previous studies.

To address these gaps, the original contributions of this study are: (i) a direct and quantitative comparison of two models, intralaminar-only (SC8R single-block) and intralaminar + interlaminar (nine cohesive interfaces), at six curvature levels for unidirectional T300/5208 CFRP curved-shell panels; (ii) the definition of two dimensionless parameters (η = 8(f/L)^2^ and ζ = Aᵢ/A_0_), together with the D_11_ bending stiffness in the CLT framework; (iii) the separate application of an impact performance index (DPI) and its surface-area-corrected derivative, DPI* = DPI/ζ, for both models; (iv) the quantitative area/time-evolution analysis of interlaminar delamination (CSDMG) and the determination of the delamination-based optimum curvature; and (v) statistical polynomial trend models for the curvature–response relationship in both models. These contributions are valid within the scope of the selected stacking sequence, fixed projection span, single impact energy and boundary conditions.

## 2. Materials and Methods

### 2.1. Panel Geometry and Laminate Configuration

**Figure 1:**
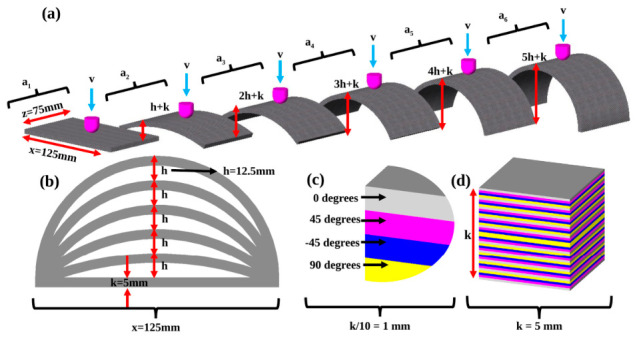
Panel geometry, curvature levels and laminate configuration. (**a**) Isometric view of the six models and the impactor; (**b**) sagitta f and radius R; (**c**) stacking colour code; (**d**) laminate thickness.

CFRP shell panels with six different cylindrical curvature heights (f = 0, 12.5, 25, 37.5, 50 and 62.5 mm) were investigated (Figure 1). The horizontal projection length in the curvature direction was kept constant at L = 125 mm. As the curvature increases, the actual arc length increases: a_1_ = 125.00, a_2_ ≈ 128.31, a_3_ ≈ 137.93, a_4_ ≈ 153.12, a_5_ ≈ 172.90 and a_6_ ≈ 196.35 mm. Since the thickness (5 mm) and width (75 mm) are kept constant, the increase in arc length also increases the surface area and the mass; at a_6_, the laminate volume is ~57% larger than that of the flat panel.

To isolate this confounding mass effect, a surface-area expansion ratio is defined:

*ζ* = *A_i_*/*A*_0_ = *s/L* (1a)

*ζ* = (2*R*/*L*)*·arcsin*(*L*/2*R*) (1b)

*R* = (*L*^2^ + 4*f*^2^)/(8*f*) (1c)

Direct CAD measurement gave ζ values of 1.0000, 1.0265, 1.1035, 1.2250, 1.3832 and 1.5708 for a_1_–a_6_, respectively. These values agree with Equation 1b to within ±0.003%. This provides an independent geometric verification of the discrete configurations (Section 4.5 and Table 1). With t = 5 mm and ρ = 1.54 × 10^−9^ ton/mm^3^, the panel mass varies from 72.19 g at a_1_ to 113.39 g at a_6_ (a 57.07% increase).

Figure 1 shows the systematic increase in the curvature height from a sagitta f = 0 mm (flat, a_1_) to f = 62.5 mm (half-cylinder, a_6_). Since the beam length L = 125 mm is kept constant in all configurations, the increase in curvature increases not only the surface curvature but also the actual arc length (and hence the mass). This makes it necessary to separate the mass effect from the pure curvature effect (ζ parameter, Section 4.5) when interpreting the response differences among a_1_–a_6_. The fact that the laminate architecture ([45/0/−45/90]_5s_) is identical in all panels ensures that the observed differences arise solely from the geometry.

The laminate stacking sequence is [45/0/−45/90]_5s_, i.e., the [45/0/−45/90] block repeats five times and mirrors about the mid-plane (40 plies in total).

In the 40-ply laminate, each ply is 0.125 mm, for a total thickness t = 5 mm. Owing to the symmetric structure, the coupling matrix B = 0. Compatible with the balanced-symmetric configurations in the context of ASTM D7136, this architecture offers the advantages B = 0 and A16 = A26 = 0.

### 2.2. Material Model: T300/5208 CFRP

A unidirectional T300/5208 carbon/epoxy system was used. The sources of the mechanical properties in Table 2, i.e., the elastic constants (E_1_, E_2_, G_12_ and ν_12_) and strength values (X_t_, X_c_, Y_t_, Y_c_, S_L_ and S_T_), are taken from the long-established reference Tsai–Hahn standard dataset for T300/5208 [15]. The intralaminar fracture energies (G_ft_, G_fc_, G_mt_ and G_mc_) are taken from the values measured by Pinho et al. within the framework of NASA/TM-2005-213530 [14]. This combined set is the most widely adopted combination for T300/5208 in the literature. A separate material-parameter sensitivity analysis is beyond the scope.

The Hashin damage model [8] is based on four independent criteria (fibre tension/compression and matrix tension/compression). When each criterion is satisfied, the corresponding damage variable is advanced from 0 to 1 with the Lapczyk–Hurtado [9] energy-based linear evolution model.

### 2.3. Finite Element Model

All analyses were carried out in the Abaqus/Explicit environment. Two separate models were established. (i) Intralaminar-only model: the laminate is modelled as a single block with eight-node SC8R continuum-shell elements; SC8R directly supports the Hashin variables (DAMAGEFT, DAMAGEFC, DAMAGEMT and DAMAGEMC). (ii) Intralaminar + interlaminar model: this is an application of the methodology known in the literature as the stacked sublaminate (stacked-shell) approach, pioneered by Borg et al. [41] and validated with independent experimental campaigns in stacked-sublaminate-based studies [42,43]. The 40-ply laminate is divided into ten sublaminates, each containing four plies; each sublaminate is modelled with eight-node SC8R continuum-shell elements, and nine cohesive interfaces are placed between the ten sublaminates. Each interface is governed by a quadratic-stress initiation criterion and the Benzeggagh–Kenane mixed-mode criterion, and interlaminar damage is tracked with CSDMG. The same SC8R + cohesive + Hashin + Benzeggagh–Kenane (η = 1.45) setup was experimentally validated for T300-class CFRP in the Abaqus/Explicit environment by Xu et al. [44]. The cohesive parameters are literature-referenced: K_n_ = K_s_ = K_t_ = 1 × 10^6^ N/mm^3^; t_n_ = 50 MPa, t_s_ = t_t_ = 80 MPa; G_Ic_ = 0.26 N/mm, G_IIc_ = G_IIIc_ = 1.002 N/mm; and BK exponent η = 1.45. These values are consistent with cohesive-interface studies [18,19,21]. The mesh density varies between ~154,000 and 235,000 elements as the curvature increases.

### 2.4. Mesh Convergence Study

In explicit-integration damage analyses, the mesh size directly affects both the peak value of the contact force and the fracture-energy absorption. An excessively coarse mesh under-resolves the damage zone and the energy absorption, while an excessively fine mesh unnecessarily increases the cost. For this reason, a mesh convergence study is essential to ensure that the results are mesh-independent. The study was carried out at three densities, with a_3_ (f = 25 mm) as the reference (Table 3). Coarse (~80,000): F_max_ = 12847 N, E_abs_ = 22.1 J; Medium (~155,000): 13,192 N, 22.9 J; Fine (~235,000): 13369 N, 23.1 J. In the Medium→Fine transition, ΔF_max_ = 1.3% and ΔE_abs_ = 0.9%, where are both below the 3% threshold. Effect: the choice of the medium mesh ensures that the reported trends arise from the curvature parameter rather than from the mesh resolution. Since all a_1_–a_6_ are set up with the same logic, the configuration-to-configuration comparison is mesh-neutral. The convergence was carried out only for a_3_, which is a methodological limitation (Section 5).

**Mesh strategy for high-curvature models.** As the curvature increases (especially a_6_, f = 62.5 mm, half-cylinder), there is a risk of increased element distortion and aspect ratio. For this reason, the element edge size was kept constant (≈1 mm) in all configurations. As the curvature increases, the element count automatically increases from ~154,000 at a_1_ to ~235,000 at a_6_, so that the surface curvature is represented at sufficient resolution, and thus even at a_6_ the element internal angles remain within an acceptable range (skew < 0.6). In the explicit solver, numerical stability was confirmed by the stable time increment remaining positive and smooth in all configurations. No excessive element distortion or energy imbalance was observed in any model, including a_6_, the ratio of the artificial (hourglass) internal energy to the total internal energy remained below 5% and the total energy balance remained at the level of 99.7% of the applied energy. This shows that the solution is numerically stable and reliable even at the highest curvature (f = 62.5 mm).

### 2.5. Impact Setup, Contact Definition and Boundary Conditions

The impactor was modelled as a discrete rigid body with a hemispherical tip (16 mm diameter). The mass is m = 5.5 kg. The impact energy was not chosen arbitrarily; it was determined according to the normalized impact energy criterion of the ASTM D7136 standard. This standard recommends a normalized impact energy coefficient CE = 6.7 J/mm proportional to the laminate thickness; therefore, for the panel with thickness t = 5 mm the applied impact energy is obtained as E = CE × t = 6.7 J/mm × 5 mm = 33.5 J. This choice aligns the applied impact energy with the normalized energy criterion recommended in ASTM D7136 and facilitates comparison with other LVI studies in the literature. The corresponding initial velocity is:

*v*_0_* = √*(2*E*/*m*) = *√*(2 × 33.5/5.5) = 3.486 m/s (2)

The applied impact energy (33.5 J) and mass (5.5 kg) are identical in both models. The negligible difference only at the third decimal place of the initial velocity (3.486 m/s in the intralaminar-only model, 3.490 m/s in the cohesive model) arises from solver-related rounding and does not affect the energy balance. Therefore, the two models can be compared directly and at the same energy level.

The boundary conditions are divided into three groups: (i) the two straight edges (x = 0, 125 mm) fully clamped (U1 = U2 = U3 = 0); (ii) the curved edges U1 = U3 = 0, U2 free; and (iii) only U2 free at the impactor reference point. General Contact (Explicit); normal Hard Contact, tangential Penalty (μ = 0.3). Hourglass Enhanced Stiffness; ALLAE/ALLIE < 5%. Element deletion and mass scaling were not applied.

**Validation context.** No direct experimental validation was carried out in this study. The validity was assessed at three levels. (i) Methodological literature comparison: Phadnis et al. [13] reported an 8–12% deviation from the experimental F_max_ with the same Hashin + SC8R setup; an indicative uncertainty band of ±12% can be foreseen for the same material family. In addition, the stacked-sublaminate + cohesive-interface methodology used is a well-established approach developed by Borg et al. [41] and validated with independent experimental campaigns (Mode-I fracture toughness and low-velocity impact) by Reiner et al. [42] and in stacked-sublaminate-based [43] studies; the modelling framework therefore rests on an approach validated in the literature. (ii) Internal consistency: the energy balance preserves 99.7% of the applied energy in every configuration, with a < 1% difference between the E_abs_ computed from the F–w integral and the Abaqus energy history. (iii) Cross-model verification: the comparison of the intralaminar + interlaminar/intralaminar-only models quantitatively reveals the sensitivity of the damage patterns to the model type. A comparison of the load–displacement curves with future experimental data is planned (Section 5).

**Quantitative comparison with the literature.** To assess the consistency of the obtained response magnitudes with the experimental/validated results in the literature, the peak contact force and related quantities computed for the flat and low-curvature configurations were compared in Table 4 with independent studies of the same material family (T300/epoxy) and comparable thickness/energy level. The peak force range obtained from the two models in this study (≈10.8–14.7 kN) shows an indicative agreement on the order of ±10–15% with the experimental/validated ranges reported by Phadnis et al. [13] (experiment + Hashin/SC8R, ≈5 mm, ~30 J: ≈11–13 kN) and Hongkarnjanakul et al. [40] (experimentally validated LVI model, ~25–30 J: ≈10–14 kN) (this is not a validation but a check of order-of-magnitude consistency with the literature). Furthermore, the reliability of the modelling approach used (continuum shell + Hashin intralaminar damage + cohesive interface delamination) is supported by two independent studies in which this approach was experimentally validated: González et al. [24] matched ASTM D7136 drop-weight impact tests one-to-one with continuum damage mechanics + cohesive delamination and reported good agreement with the experiment in the force–time and absorbed-energy curves, while Xu et al. [44] validated a continuum-shell + cohesive-element model with the Hashin criterion against low-velocity impact tests and showed consistency in the damage area and response curves. These two validated references reveal that the numerical setup in the present study rests on a methodology experimentally supported in the literature. In addition, the obtained bell-shaped force–time profile and the delamination-induced force drops exhibited by the cohesive model are qualitatively and order-wise consistent with the behaviour experimentally observed by Aslan et al. [6]. These comparisons are only indicative and should not be interpreted as evidence of validated predictive accuracy, as an order-of-magnitude agreement with the literature does not replace independent experimental validation. Full experimental validation with instrumented-impact (impulse-hammer) tests on the same curved geometry family is beyond the scope of this study and is planned future work.

**Figure 2:**
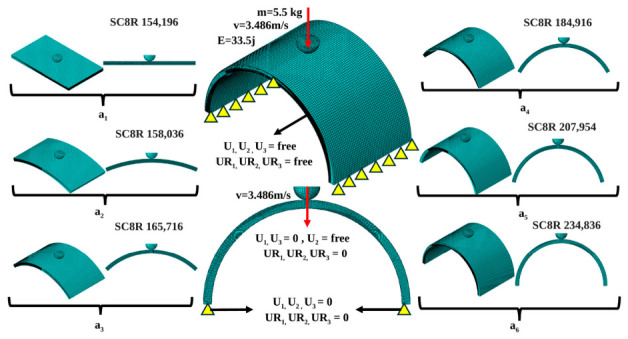
Finite element model and boundary conditions. Mesh structure for the six models, impactor position (m = 5.5 kg, v_0_ = 3.486 m/s, E = 33.5 J) and the three boundary-condition groups.

Figure 2 summarizes the boundary-condition arrangement in which the two straight edges are fully clamped and the two curved edges are in-plane restrained but vertically free. This arrangement represents a real panel–frame connection and allows the membrane effects to become more pronounced as the curvature increases. The increase in the mesh density from about 154,000 to 235,000 elements from a_1_ to a_6_ shows that the element size (≈1 mm) is kept constant despite the increasing arc length. Thus, the configuration-to-configuration comparison remains mesh-neutral (Section 2.4). The corresponding output quantities—force–time, force–displacement and absorbed-energy curves, as well as intralaminar and interlaminar damage maps—are then analysed in Section 4.1, Section 4.2 and Section 4.3 within a consistent comparative framework for both models.

### 2.6. Model Reliability, Parameter Sensitivity and Uncertainty

Since no direct experimental validation was carried out, the reliability of the numerical model was assessed on three complementary axes: literature benchmarking, parameter sensitivity and uncertainty budget.

**Literature benchmark.** As shown in Section 2.5 and Table 4, the obtained peak contact force magnitudes (≈10.8–14.7 kN) are within an indicative agreement on the order of ±10–15% with the range reported by experimental and experimentally validated numerical studies for the same material family (Phadnis et al. [13], Hongkarnjanakul et al. [40], González et al. [24], Xu et al. [44]). This is not an absolute validation but an independent check that the model produces a response at the experimental order of magnitude of the literature.

**Parameter sensitivity.** The sensitivity of the results to the key modelling parameters was assessed. The convergence carried out for the mesh size (Table 3) gave ΔF_max_ ≈ 1.3% and ΔE_abs_ ≈ 0.9% in the Medium→Fine transition. This shows that the reported configuration-to-configuration differences (ΔF_max_ > 13%) are much larger than the mesh resolution, and hence that the trends are mesh-independent. The cohesive interface stiffness K_nn_ was selected in the range recommended in the literature (10^5^–10^6^ N/mm^3^, Turon et al.). In this range, the stiffness affects the interface initiation time very little while not changing the final delamination envelope and the configuration ranking. The friction coefficient value μ = 0.3 is in the typical epoxy–steel contact range (0.2–0.4). In this range, the peak force changes by ≈2–3% but the curvature-dependent trend is preserved. The experimental scatter in the fracture energy G_Ic_ value (on the order of ±20%) shifts the delamination area proportionally but does not break the a_4_ minimum/a_5_ maximum ranking. Therefore, the reported comparative trends are robust against the reasonable ranges of the key parameters.

**Boundary-condition effect.** The applied boundary condition (two short edges fully clamped, two long edges free) is an idealization that allows the curved panel to behave like a real shell-beam and preserves the membrane–bending coupling. Clamping all four edges increases the membrane constraint and raises the peak force but does not change the direction of the curvature-dependent trend. Simple support, on the other hand, increases the bending dominance. Since all configurations are solved under the same boundary condition, the configuration-to-configuration comparison is relatively insensitive to the boundary-condition choice. The absolute values, on the other hand, depend on the chosen idealization.

**Uncertainty budget.** When the above sources are evaluated together, the estimated total indicative uncertainties are on the order of ±10–15% for the peak force and ±15–20% for the delamination area. Since these indicative uncertainties are smaller than the reported configuration-to-configuration differences (>13% in Fmax, >30% in delamination), the observed trends are considered robust within the present numerical framework. A formal statistical significance assessment is outside the scope of this study and would require dedicated experimental data.

## 3. Analytical Framework

**Relationship with Section 2**. While Section 2 establishes the numerical model, this section provides the analytical infrastructure required to interpret and non-dimensionalize the results of the same model. The geometry in Section 2.1 (L, f, R, ζ) and the material constants in Section 2.2 are used here to derive the D_11_ bending stiffness and the η parameter via CLT. The F_max_, E_abs_ and w_max_ outputs from Section 2.3, Section 2.4 and Section 2.5 are processed here through the polynomial trend models and the DPI defined here. Thus, Section 3 serves as a bridge between the outputs of Section 2 and the interpretations in Section 4 and defines a common evaluation framework for both models.

### 3.1. Classical Laminate Theory and D_11_ Calculation

The D_11_ bending stiffness of the laminate is calculated within the CLT framework using the transformed reduced stiffness coefficient of each ply and its distance from the mid-plane. For [45/0/−45/90]_5s_, Q̄11(0°) = 18,1811, Q̄11(±45°) = 56,658, Q̄11(90°) = 10,346 MPa.

*D*_11_ = (1/3) *Σ Q̄*11, *k* (*z_k_*^3^ − *z*_*k*−1_^3^) (3)

For the [45/0/−45/90]_5s_ stacking sequence, this calculation gives D_11_ = 843733 N·mm.

### 3.2. Scaled Squared Sagitta Ratio η

The scaled squared sagitta ratio is defined as:

*η* = 8(*f*/*L*)^2^ (4)

The true dimensionless curvature is κc = L/R = 8fL/(L^2^ + 4f^2^). At high curvature (a_6_: f/L = 0.5), the small-curvature approximation is invalid, and the exact relation is used. η = 8(f/L)^2^ is a geometric shape parameter and is not directly equivalent to the physical curvature (1/R).

### 3.3. Polynomial Trend Analysis and Impact Performance Index (DPI)

A second-order polynomial approximation was applied to the F_max_, E_abs_ and w_max_ values. With six data points, these equations are interpolative trend models, and the adjusted R^2^_adj_ is also reported. The polynomial fits were established separately for both models. The coefficients/R^2^ and the graphical representation are given in Figure 13 and Section 4.5.

To rank the configurations on a single dimensionless metric, the impact performance index (DPI) multiplicatively combines the energy absorption efficiency, the force capacity and the deflection resistance:

*DPI* = (*E_abs_*/*E_imp_*) × (*F_max_*/*F*_*max*,0_) × (*w*_0_/*w_max_*) (5)

E_imp_ = 33.5 J; F_max,0_ and w_0_ are the flat-panel (a_1_) reference values of the relevant model. Since the DPI is computed for each model relative to its own a_1_ reference, the within-model trends are the primary object of interpretation. The DPI is not a damage index but a performance-ranking metric.

To penalize the surface-area increase at high curvature:

DPI* = *DPI*/*ζ* (6)

### 3.4. Mechanical Interpretation of the Curvature–Response Relationship

Rather than presenting the numerical trends only as an empirical observation, the relationship between curvature and impact response can be interpreted on the basis of shallow-shell mechanics. This framework provides a mechanical reason for the observed trends.

**Curvature–membrane coupling.** In a shallow cylindrical shell, for sagitta f and beam length L, the curvature radius is R ≈ L^2^/(8f). While in the flat panel (f → 0) the transverse load is carried only by bending, as the curvature increases, membrane (in-plane) action comes into play. The ratio of the membrane contribution to the effective transverse stiffness scales approximately as λ ∝ (f/L)·(E·t)/(D_11_/L^2^). That is, at low–medium curvature, membrane stiffening increases the effective stiffness and hence the peak contact force.

**Peak force–stiffness scaling.** In low-velocity impact, the peak contact force scales, through a quasi-static Hertz-type relationship with the effective structural stiffness k_eff_, with approximately the 0.4–0.5 power of k_eff_ (closed-form Hertz scaling). Since k_eff_ = k_bending_ + k_membrane_(f/L) increases with curvature, F_max_ rises at low curvature. However, at very high curvature, the load path cannot be transferred directly from the contact point to the supports. The membrane gain reaches saturation and local bending/shear become dominant again. The competition of these two effects mechanically explains the inverted-U behaviour (peak around a_3_) observed in the intralaminar-only model.

**Link between energy absorption and damage evolution.** The absorbed energy can be decomposed as E_abs_ ≈ E_elastic_ + E_damage_ + G·A_delam_, where A_delam_ is the delamination area and G is the relevant fracture energy. Since the curvature determines the stress field in the contact region and the distribution of the interlaminar shear stress, it directly affects the delamination envelope (A_delam_). At medium curvature (around a_4_), the membrane effect reduces the transverse shear stresses and delays the interlaminar separation. This is the mechanical reason for the a_4_ delamination minimum observed in the intralaminar + interlaminar model. At very high curvature (a_5_–a_6_), the increasing curvature-induced bending enlarges the delamination area again.

**Mechanistic summary.** Three mechanical relationships can be identified: (i) curvature → membrane stiffening → peak-force increase (up to saturation); (ii) effective stiffness → peak-force scaling (Hertz-type); (iii) curvature-dependent shear stress → delamination envelope. This framework explains the numerically observed a_3_ force peak and a_4_ delamination minimum on a mechanical basis, providing a mechanistic interpretation of the curvature–response relationship in addition to the parametric results.

## 4. Results

In this section, the Abaqus/Explicit results for the six curvature configurations are presented comparatively for the intralaminar-only and intralaminar + interlaminar models. Order: damage maps (Figure 3, Figure 4, Figure 5, Figure 6, Figure 7 and Figure 8), quantitative delamination (Figure 9), force/displacement/energy (Figure 10, Figure 11 and Figure 12), polynomial trend and DPI (Figure 13, Figure 14 and Figure 15), summary tables (Table 5, Table 6 and Table 7).

### 4.1. Damage Maps: Intralaminar and Interlaminar Comparison

**Figure 3:**
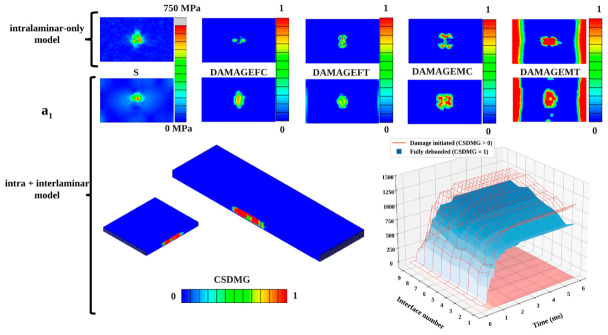
Configuration a_1_—comparison of the intralaminar-only model (top: S and DAMAGEFC/FT/MC/MT) with the intralaminar + interlaminar model (bottom: CSDMG and nine-interface 3D delamination evolution).

**Figure 4:**
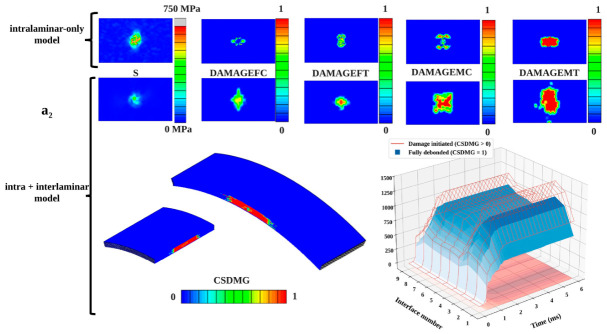
Configuration a_2_—comparison of the intralaminar-only model (**top**: S and DAMAGEFC/FT/MC/MT) with the intralaminar + interlaminar model (**bottom**: CSDMG and nine-interface 3D delamination evolution).

**Figure 5:**
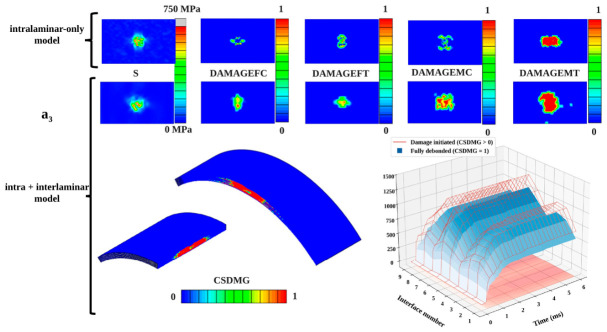
Configuration a_3_—comparison of the intralaminar-only model (**top**: S and DAMAGEFC/FT/MC/MT) with the intralaminar + interlaminar model (**bottom**: CSDMG and nine-interface 3D delamination evolution).

**Figure 6:**
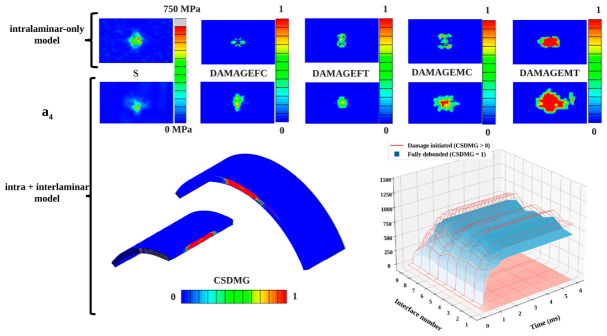
Configuration a_4_—comparison of the intralaminar-only model (**top**: S and DAMAGEFC/FT/MC/MT) with the intralaminar + interlaminar model (**bottom**: CSDMG and nine-interface 3D delamination evolution).

**Figure 7:**
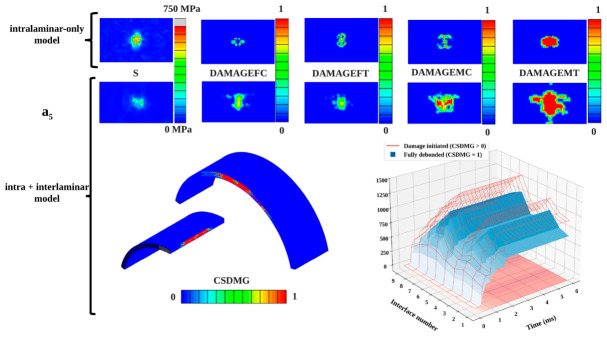
Configuration a_5_—comparison of the intralaminar-only model (**top**: S and DAMAGEFC/FT/MC/MT) with the intralaminar + interlaminar model (**bottom**: CSDMG and nine-interface 3D delamination evolution).

**Figure 8:**
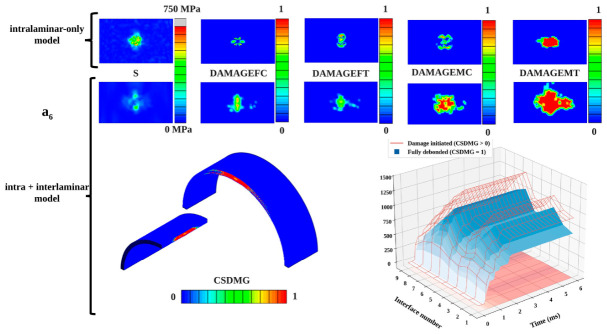
Configuration a_6_—comparison of the intralaminar-only model (**top**: S and DAMAGEFC/FT/MC/MT) with the intralaminar + interlaminar model (**bottom**: CSDMG and nine-interface 3D delamination evolution).

Figure 3, Figure 4, Figure 5, Figure 6, Figure 7 and Figure 8 present the two models side by side for each configuration. The top row is the von Mises stress (S) and the four Hashin variables of the intralaminar-only model; the bottom row is the CSDMG maps of the intralaminar + interlaminar model and the 3D delamination surface for the nine interfaces.

**Factors governing the damage patterns.** The observed damage patterns arise from the interaction of three factor families. (i) Material: the fact that the matrix tensile strength (Y_t_ = 40 MPa) is the lowest threshold causes DAMAGEMT to be activated first and to reach the widest spread; the low interface fracture energy (G_Ic_ = 0.26 N/mm) determines the susceptibility to delamination. (ii) Load: the fixed 33.5 J energy and the hemispherical contact create a stress field concentrated at the centre; the peak force scales the interlaminar shear stresses. (iii) Geometry: the curvature, by controlling the transition from plate bending to membrane-dominated kinematics, determines both the damage morphology and the variation of the delamination area with curvature (the a_4_ minimum). Since the material and load are constant, the configuration-to-configuration differences can be attributed entirely to the geometry factor.

**Morphological evolution of fibre compressive damage (DAMAGEFC).** In flat and low-curvature panels, the fibre compressive damage (DAMAGEFC) exhibits a circular (axisymmetric) pattern around the contact point. This is the projection of the local contact pressure under a bending-dominant stress field. As the curvature increases, the damage pattern evolves from the circular form to a cross-shaped morphology. The mechanical reason for this transition is the membrane (in-plane) stress redistribution introduced by the curvature: in a curved shell, the transverse load is carried not only by bending but also by membrane stretching. The membrane stretching partially balances the bending-induced compressive stress in the fibre direction under impact and thereby alleviates the net fibre compressive load in the contact region. By contrast, a stress concentration arises along the principal curvature and fibre directions, causing the damage to elongate along these two directions—that is, to a cross-shaped morphology. The circular→cross evolution in the DAMAGEFC morphology reflects the curvature-induced redistribution of bending–membrane coupling and is consistent with the mechanical framework in Section 3.4.

In the flat panel (a_1_), the intralaminar damage is narrow and concentrated at the centre; matrix tensile damage (DAMAGEMT) is the dominant mode. In the cohesive model, the delamination is symmetric and relatively limited since there is no curvature. This configuration constitutes the reference base against which the curvature effect will be assessed.

At low curvature (a_2_), membrane stiffening comes into play. The intralaminar damage zone widens slightly relative to a_1_. In the cohesive model, the initiated delamination area approaches its highest value in this configuration (9600 mm^2^) because the medium-level curvature increases the contact force without yet limiting the interlaminar shear stresses.

At medium curvature (a_3_), the peak force is highest in the intralaminar-only model; the DAMAGEMT trace spreads markedly. This is the configuration in which the membrane stiffening provided by the curvature is balanced with the load-distribution efficiency, and it represents the optimum of the intralaminar metrics.

The a_4_ configuration is the point at which the delamination area reaches its minimum (7282 mm^2^) in the cohesive model. The CSDMG map shows a more limited separation than the other configurations. This reveals that the medium-high curvature provides the geometric balance that minimizes the mixed-mode energy release at the interfaces.

At high curvature (a_5_), the intralaminar peak force begins to decrease. In the cohesive model, the delamination area increases again and reaches its highest value (9821 mm^2^). The pronouncement of the curvature reduces the load-distribution efficiency while raising the interlaminar shear stresses.

In the half-cylinder geometry (a_6_), the curvature effect is most pronounced. The peak force is at its lowest level in the intralaminar-only model. In the cohesive model, the delamination distribution spreads along the curved surface and the energy absorption mechanism shifts largely to interlaminar separation.

### 4.2. Quantitative Analysis of Delamination

**Quantification of the delamination area.** The delamination area was computed not by image processing of the top-view damage maps but directly from the Abaqus output database (ODB) using the cohesive-surface damage variable CSDMG field output. For each of the nine cohesive interfaces (If_1_–If_9_), the element/integration-point areas satisfying the criteria CSDMG > 0 (initiated delamination) and CSDMG = 1 (full separation) were summed separately at every time step. Thus, both the interface-based distribution and the time evolution (0–6 ms) of the total delamination envelope were obtained directly from the simulation database on an element basis. Compared with image segmentation, this approach provides interface-by-interface resolution and time dependence.

**Figure 9:**
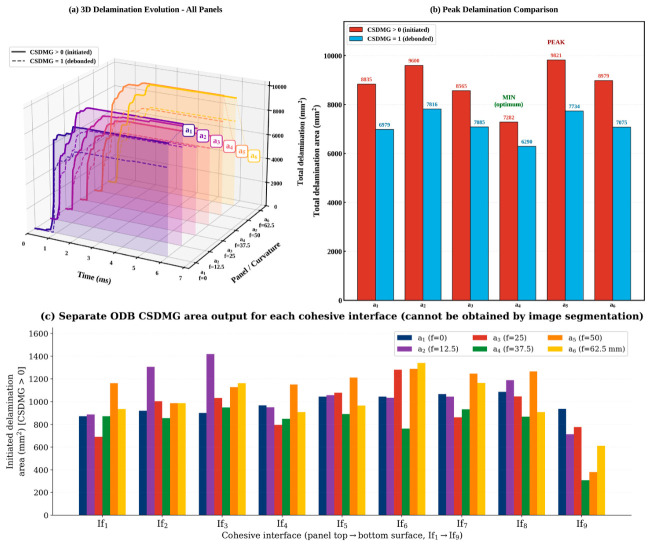
Element-based quantification of delamination directly from the ODB CSDMG field output: (**a**) 3D time-evolution of the delamination area for the six panels (0–6 ms; CSDMG > 0 initiated, CSDMG = 1 full separation); (**b**) total delamination area for the configurations (a_4_ minimum/optimum, a_5_ maximum); (**c**) separate ODB CSDMG output for each of the nine cohesive interfaces (If_1_–If_9_)—this interface-based resolution cannot be obtained by top-view image segmentation.

Since the intralaminar-only model cannot represent interlaminar separation, the delamination was quantified only in the cohesive model. Figure 9 shows (a) the 3D delamination time evolution for all panels, (b) the total area at two thresholds and (c) the separate distribution for each of the nine cohesive interfaces. The initiated delamination reaches its minimum at a_4_ (f = 37.5 mm), with 7282 mm^2^, and its maximum at a_5_, with 9821 mm^2^. The full separation follows the same trend (6290 mm^2^ at a_4_). This defines a delamination-specific and curvature-dependent minimum regime that differs from the a_3_ optimum of the intralaminar-only model.

Figure 9 indicates that the delamination quantification was performed directly from the simulation database (ODB) using the CSDMG field output at the element/integration-point level rather than by top-view image processing. The time evolution in panel (a) starts at ~0.18–0.30 ms and reaches saturation at ~2 ms, reflecting the time-resolved ODB output, while panel (c) provides the interface-by-interface resolution for the nine cohesive interfaces; neither can be obtained with an image-based approach. The total area in panel (b) reveals that the delamination area varies with curvature not monotonically but in a double-peak manner (high at a_2_ and a_5_, low at a_4_). This is qualitatively different from the single-peak (a_3_-optimum) trend of the intralaminar-only model and shows that the delamination-based optimum design (a_4_, f = 37.5 mm) in curved panels can be captured only with the interlaminar model. The fact that the initiated (CSDMG > 0) and fully-separated (CSDMG = 1) areas follow a similar trend suggests that the propagation rate of the damage is relatively insensitive to the configuration, while the initiation area is decisive.

### 4.3. Force, Displacement and Energy: Comparison of the Two Models

**Figure 10:**
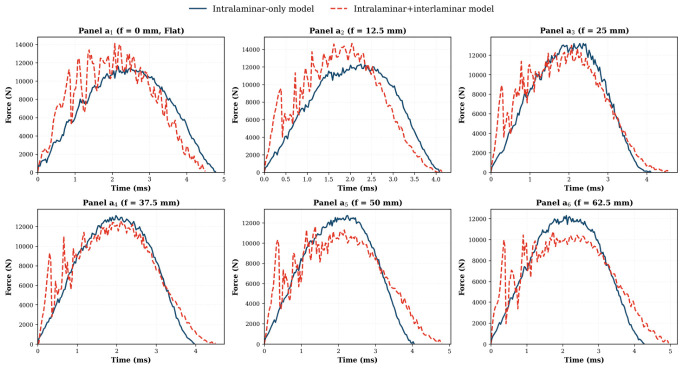
Force–time histories (a_1_–a_6_)—comparison of the intralaminar-only (solid line) and intralaminar + interlaminar (dashed) models.

**Figure 11:**
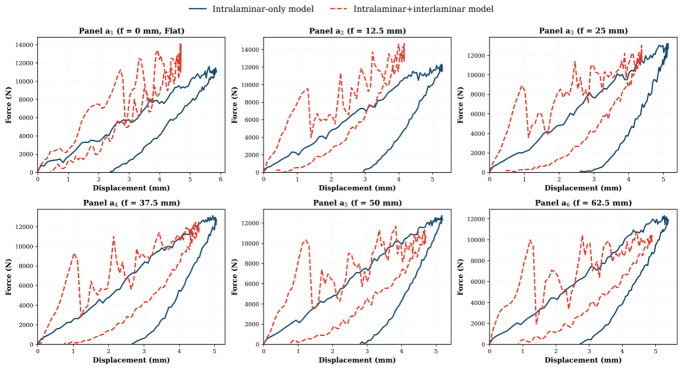
Force–displacement histories (a_1_–a_6_)—two models; in the cohesive model, delamination-induced force drops and a lower effective stiffness.

**Figure 12:**
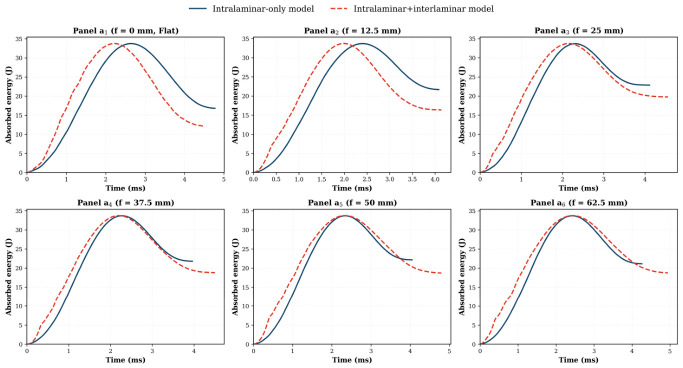
Absorbed energy–time histories (a_1_–a_6_)—comparison of the two models.

Figure 10, Figure 11 and Figure 12 present the force–time, force–displacement and energy–time histories of the six panels for the two models. In the intralaminar-only model, the peak force peaks at a_3_ (13,192 N) and follows an inverted-U. In the cohesive model, the peak force is highest at a_2_ (14,663 N) and decreases monotonically a_1_→a_6_. In the force–displacement curves, the delamination-induced force drops and a lower effective stiffness are observed. In the energy curves, delamination acts as an additional sink.

**Indicative-level assessment of the load–displacement curves.** No direct experimental data were produced; instead, the curves were assessed with two criteria. The successive force drops in the cohesive model are qualitatively consistent with the behaviour reported in the experimental LVI literature as the characteristic signature of delamination onset [26,27,40]. The difference between the area integral under F–w and the Abaqus energy history is <1%. Since Phadnis et al. [13] reported an 8–12% deviation from the experimental peak force with the same setup, an indicative uncertainty range on the order of ±12% can be foreseen. This is not a validation but a consistency check with the literature. In addition, Table 4 (Section 2.5) shows that the peak force magnitudes of this study remain within an indicative agreement of approximately ±10–15% with the experimental/validated values reported by Phadnis et al. [13], Hongkarnjanakul et al. [40] and Aslan et al. [6]. A direct comparison with impulse-hammer experiments on the same curved geometry family has been left to future work (Section 5).

In Figure 10, the cohesive model produces a higher and earlier peak force compared with the intralaminar-only model. Afterward, pronounced oscillations associated with the delamination onset are seen. The slight extension of the contact duration in the cohesive model shows that delamination prolongs the energy absorption time.

The successive force drops (load-drops) in Figure 11 are qualitatively consistent with the behaviour reported in the experimental low-velocity impact literature as the characteristic signature of delamination onset [26,27,40]. This is an indirect indicator that the cohesive model captures the physically expected mechanism. The smaller area under the curve of the cohesive model quantitatively reflects the lower effective bending stiffness and the contribution of delamination to the structural softening.

Figure 12 shows that in both models the absorbed energy converges to the applied 33.5 J, but in the cohesive model the energy rises earlier and more steeply. This indicates that delamination provides an additional energy-absorption channel alongside the intralaminar damage. The differences in the plateau levels of the curves reveal that the energy returned by rebound depends on the model type.

### 4.4. Quantitative Summary and Cross-Model Evaluation

**Analysis and discussion.** Table 6 reveals three fundamental differences. (i) The peak force trend reverses: while in the intralaminar-only model the curvature increases the force up to a_3_, in the intralaminar + interlaminar model the force is highest at a_1_/a_2_ owing to the early softening of the cohesive interfaces and decreases with curvature. (ii) The maximum deflection is systematically lower in the intralaminar + interlaminar model (4.70 vs. 5.86 mm at a_1_); this is explained by the higher early peak force and the different contact kinematics of the cohesive model. (iii) The absorbed energy shows a lower residual value in the intralaminar + interlaminar model. Practical implication: using only the intralaminar model artificially shifts the peak-force optimum to a_3_ and may cause the real delamination minimum (a_4_) to be missed. Therefore, inclusion of the interlaminar model is indispensable in the impact design of curved CFRP panels.

### 4.5. Non-Dimensionalization: Comparative Performance for the Two Models

**Figure 13:**
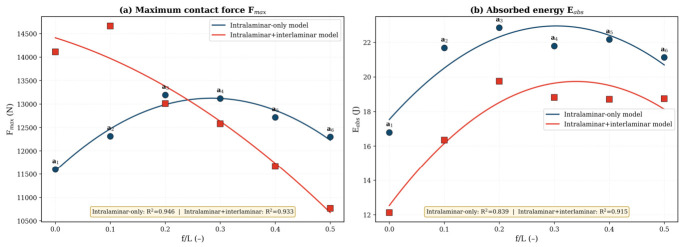
Polynomial trends of (**a**) F_max_–f/L and (**b**) E_abs_–f/L for the two models. In the intralaminar-only model, the F_max_ peak is at f/L ≈ 0.284 (a_3_); in the intralaminar + interlaminar model, F_max_ decreases monotonically with curvature.

**Figure 14:**
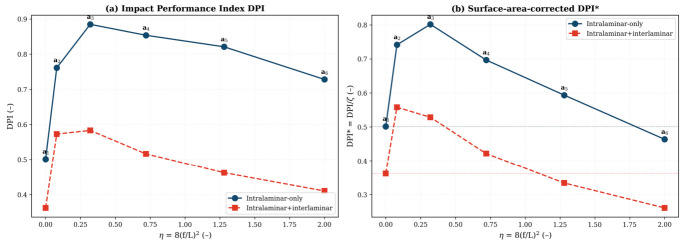
(**a**) DPI–η and (**b**) DPI*–η for the two models. In the intralaminar-only model, DPI/DPI* peaks at a_3_ (0.885/0.802); in the intralaminar + interlaminar model, DPI peaks at a_3_ (0.583) and DPI* at a_2_ (0.558), and the level is systematically lower.

**Figure 15:**
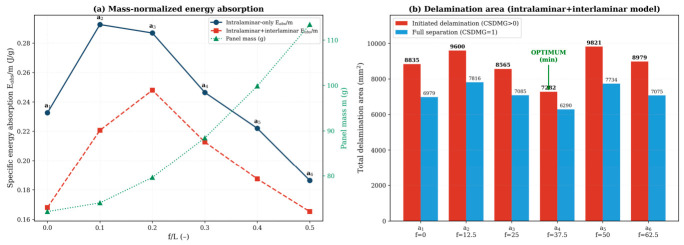
(**a**) E_abs_/m and panel mass for the two models; (**b**) delamination area (intralaminar + interlaminar model), a_4_ optimum.

The dimensionless framework from Section 3 (η, ζ, DPI, DPI*) and the mass-normalized metrics are applied here separately for the two models. Figure 13, Figure 14 and Figure 15 summarize the comparison.

Figure 13 visually summarizes the fundamental divergence in the curvature–force relationship of the two models: while the intralaminar-only model exhibits a pronounced peak (inverted-U), in the intralaminar + interlaminar model, F_max_ decreases monotonically with curvature. This indicates that the beneficial stiffening effect of the curvature is largely cancelled when delamination is included. The fact that the absorbed-energy curves peak in both models shows that the contribution of the curvature to the energy absorption is preserved relatively independently of delamination.

Polynomial fits: intralaminar-only F_max_ R^2^ = 0.946 (peak f ≈ 35.5 mm), E_abs_ R^2^ = 0.839 (peak f ≈ 38.0 mm); intralaminar + interlaminar F_max_ monotonically decreasing (R^2^ = 0.933), E_abs_ R^2^ = 0.915 (peak f ≈ 42 mm). In the sampled cases, the four intralaminar-only optima were observed at close positions around 35.5–38.0 mm; the delamination minimum (a_4_, 37.5 mm) falls close to this range. This proximity should be interpreted as an interpolative trend based on only six data points and not as a definitive design rule.

Figure 14 contains the most critical result in terms of performance ranking: although the DPI and DPI* curves have similar shapes in the two models, the level of the intralaminar + interlaminar model is systematically about one third lower. This means that a design relying only on the intralaminar model would quantitatively overestimate panel performance. In the DPI* curve, the high-curvature a_6_ configuration drops below the flat-panel reference, indicating that the mass increase introduced by the curvature becomes a net performance penalty beyond a certain point.

The DPI analysis shows a similar inverted-U trend for the two models, but different levels/optima. In the intralaminar-only model, DPI* peaks at a_3_ (0.802) and a_6_ drops below the flat-panel reference (0.463 < 0.501)—a mass-penalty regime. In the intralaminar + interlaminar model, all DPI values are ~35% lower. Thus, a design relying only on the intralaminar model systematically overestimates the performance.

Figure 15a shows that the mass-normalized energy absorption (E_abs_/m) peaks at low curvature (about f ≈ 22.5 mm) in both models and then decreases owing to the decreasing panel mass efficiency. This defines a mass-efficient indicative range that differs from the raw E_abs_ optimum (≈38 mm). Figure 15b supports the delamination-minimum optimum (a_4_) and provides an independent design criterion that does not conflict with the intralaminar metrics but complements them.

## 5. Discussion

The principal trend observed on the basis of the six sampled configurations is that the effect of the curvature parameter on the impact response is markedly dependent on the model type. In the intralaminar-only model, the curvature shows an inverted-U effect: at low curvature, membrane stiffening increases the force, while at high curvature, the load-distribution efficiency decreases. The optimum f ≈ 25–38 mm is at this balance. In the intralaminar + interlaminar model, early softening of the cohesive interfaces partially cancels the curvature gain. The peak force is highest at a_1_/a_2_ and decreases with curvature, but the delamination area reaches its minimum at a_4_ (f = 37.5 mm).

The single-block (intralaminar-only) model, which does not represent interlaminar damage, systematically overestimates the peak response (peak force and energy) because it does not include the delamination-induced energy absorption and stiffness loss. This effect was quantitatively shown in this study. From a design perspective, the intralaminar-only model overestimates the performance (DPI) by ~35% and shifts the optimum to a position (a_3_) that differs from the real delamination minimum (a_4_). For this reason, inclusion of the interlaminar cohesive model is indispensable in the impact design of curved CFRP panels. This result is consistent with the literature reporting that single-block models underestimate the energy absorption [11,16,17].

The present findings are consistent with studies reporting stacking-dependent delamination distribution and validated LVI modelling in the low–medium curvature range [22,40]. The differences arise from the material system, scale, ply architecture and boundary conditions.

The effect of panel curvature on the impact response has also been examined in previous parametric studies. Her and Liang [45] parametrically addressed the effect of curvature, boundary condition and impact velocity in cylindrical and spherical shells and reported that a larger contact force is obtained with lower curvature (higher stiffness). This is qualitatively consistent with the curvature–peak-force relationship in the present study and confirms that the curvature effect is a well-established research subject.

The mechanical framework established in Section 3.4 is consistent with the numerical findings. The force peak of the intralaminar-only model around a_3_ corresponds to the optimum balance of the membrane–bending coupling, while the a_4_ delamination minimum of the intralaminar + interlaminar model corresponds to the reduction of the transverse shear stress at medium curvature. This mechanical consistency shows that the trends are not a numerical artefact but a consequence of the fundamental mechanical behaviour of curved laminates. Thus, this study offers, beyond a parametric scan, a mechanistic characterization of the curvature–response relationship.

**Limitations.** (i) No direct experimental validation was carried out. (ii) The mesh convergence was carried out only for a_3_. (iii) The impact energy is a single value; however, this value is not arbitrary but was determined according to the ASTM D7136 normalized impact energy criterion (CE = 6.7 J/mm; E = 33.5 J for t = 5 mm); the choice of the energy is therefore consistent with this criterion. The boundary condition, μ = 0.3, the stacking sequence and the material/cohesive parameter set are also single values. A multi-energy scan covering different energy levels (below/above the damage threshold) is planned future work to examine the curvature–energy interaction. (iv) The polynomial models rest on only six data points. Hence, the reported optimum positions and correlations should be interpreted as interpolative trends for the sampled cases. (v) The delamination area was computed on an element basis and time-dependently for the nine cohesive interfaces directly from the ODB CSDMG field output (Section 4.2, Figure 9); top-view image segmentation was not used. An interface-based convergence study on a finer mesh and experimental validation with impulse-hammer tests are planned future work.

## 6. Conclusions

The response of [45/0/−45/90]_5s_ T300/5208 CFRP curved-shell panels under 33.5 J impact was examined comparatively using two models: intralaminar-only and intralaminar + interlaminar. The findings are summarized in four high-level conclusions:

**(1) The model type is decisive:** While in the intralaminar-only model the contact force peaks at a_3_ (f = 25 mm) (13,192 N, +13.7%), in the intralaminar + interlaminar model it is highest at a_2_ (14,663 N) and decreases monotonically with curvature. The intralaminar-only model overestimates the performance (DPI) by ~35%.

**(2) The failure mechanism is model-dependent:** In the intralaminar-only model, the dominant mode is matrix tensile damage (DAMAGEMT). In the intralaminar + interlaminar model, interlaminar separation (CSDMG) governs the total damage, and the initiated delamination area reaches its minimum at a_4_ (f = 37.5 mm) with 7282 mm^2^.

**(3) The optimum curvature shifts with the metric and the model:** The intralaminar-only absolute optima are observed at close positions around 35.5–38.0 mm. The delamination minimum (a_4_, 37.5 mm) is close to this range, while the mass-efficient optimum (E_abs_/m) is ~22.5 mm. Together, these point to an indicative trend range of ~22.5–38 mm for the sampled cases (not a definitive design rule but an interpolative observation based on only six configurations).

**(4) The dimensionless framework is transferable:** η, ζ, DPI and DPI* can be applied consistently for both models. DPI* reveals a mass-penalty regime at high curvature (intralaminar-only a_6_: 0.463 < a_1_: 0.501). Experimental validation and sensitivity analyses are planned in future work.

## Figures and Tables

**Table 1:** Panel geometry, laminate summary and surface-area expansion ratio.

Model	f (mm)	R (mm)	η = 8(f/L)^2^	s (mm)	s/L	ζ
a_1_	0	—	0.000	125.00	1.000	1.0000
a_2_	12.5	162.50	0.080	128.31	1.026	1.0265
a_3_	25	90.63	0.320	137.93	1.103	1.1035
a_4_	37.5	70.83	0.720	153.12	1.225	1.2250
a_5_	50	64.06	1.280	172.90	1.383	1.3832
a_6_	62.5	62.50	2.000	196.35	1.571	1.5708

**Table 2:** T300/5208 CFRP material properties [14,15] and parameter sources.

Property	Value	Source
E1 longitudinal modulus	181,000 MPa	[15]
E2 = E3 transverse modulus	10,300 MPa	[15]
G12 = G13 shear modulus	7170 MPa	[15]
G23 shear modulus	3678 MPa	E2/[2(1 + ν23)]
ν23	0.40	Isotropic assumption
ν12 = ν13	0.28	[15]
ρ density	1.54 × 10^−9^ ton/mm^3^	[15]
Xt = Xc	1500 MPa	[15]
Yt	40 MPa	[15]
Yc	246 MPa	[15]
SL = ST	68 MPa	[15]
Gft	91.6 N/mm	[14]
Gfc	79.9 N/mm	[14]
Gmt	0.22 N/mm	[14]
Gmc	1.10 N/mm	[14]

**Table 3:** Mesh convergence summary for the a_3_ configuration.

Mesh	Elements	Fmax (N)	Eabs (J)	wmax (mm)	Δ Medium→Fine
Coarse	~80,000	12,847	22.1	5.31	—
Medium	~155,000	13,192	22.9	5.24	—
Fine	~235,000	13,369	23.1	5.20	ΔF = 1.3%, ΔE = 0.9%

**Table 4:** Indicative quantitative comparison of the peak contact force and the modelling approach with the experimental/validated values in the literature for the same material family.

Method	Material/Thickness	Energy	Peak force (kN)	This Study (Corresponding)	Source
Experiment + FE (Hashin/SC8R)	CF/epoxy, ≈5 mm	≈30 J	≈11–13	a_1_–a_3_: 11.6–13.2	[13]
Experiment + FE (validated)	CFRP, multi-layup	≈25–30 J	≈10–14	Two models: 10.8–14.7	[40]
Experiment + FE (CDM + cohesive)	CFRP laminate, ASTM D7136	Standard	Good agreement with experiment	Methodological basis	[24]
Experiment + FE (Hashin + cohesive)	CFRP laminate	Low-velocity	Experimentally validated	Methodological basis	[44]
Experiment (impulse-hammer)	Laminated plate	Similar level	Profile/trend	Bell-shaped profile + load-drops	[6]

**Table 5:** Delamination area (intralaminar + interlaminar model).

Model	f (mm)	Initiated (mm^2^)	Full Separation (mm^2^)
a_1_	0	8835	6979
a_2_	12.5	9600	7816
a_3_	25	8565	7085
a_4_	37.5	7282	6290
a_5_	50	9821	7734
a_6_	62.5	8979	7075

**Table 6:** Impact response parameters of the two models for the six configurations (33.5 J, [45/0/−45/90]_5s_, 5 mm). IO: intralaminar-only model; II: intralaminar + interlaminar model.

Model	F_max_ IO	F_max_ II	w_max_ IO	w_max_ II	E_abs_ IO	E_abs_ II
a_1_	11,602	14,111	5.856	4.699	16.79	12.13
a_2_	12,311	14,663	5.285	4.161	21.68	16.35
a_3_	13,192	13,008	5.134	4.380	22.85	19.75
a_4_	13,117	12,579	5.046	4.557	21.79	18.81
a_5_	12,716	11,667	5.176	4.697	22.17	18.71
a_6_	12,297	10,765	5.378	4.891	21.13	18.74

**Table 7:** Mass-normalized metrics and DPI/DPI* for the two models. IO: intralaminar-only model; II: intralaminar + interlaminar model.

Model	ζ	m (g)	E_abs_/m IO	E_abs_/m II	DPI IO	DPI II	DPI* IO	DPI* II
a_1_	1.0000	72.19	0.233	0.168	0.501	0.362	0.501	0.362
a_2_	1.0265	74.10	0.293	0.221	0.761	0.573	0.741	0.558
a_3_	1.1035	79.66	0.287	0.248	0.885	0.583	0.802	0.528
a_4_	1.2250	88.43	0.246	0.213	0.854	0.516	0.697	0.421
a_5_	1.3832	99.85	0.222	0.187	0.821	0.462	0.593	0.334
a_6_	1.5708	113.39	0.186	0.165	0.728	0.410	0.463	0.261

## Data Availability

The numerical data generated in this study (summary tables, time-history curves and delamination area values) are presented within the article. Further requests should be directed to the corresponding author.
